# Low-Intensity Light-Responsive Anticancer Activity of Platinum(II) Complex Nanocolloids on 2D and 3D In Vitro Cancer Cell Model

**DOI:** 10.1155/2022/9571217

**Published:** 2022-04-23

**Authors:** Viviana Vergaro, Francesca Baldassarre, Federica De Castro, Danilo Migoni, Maria Michela Dell'Anna, Piero Mastrorilli, Francesco Paolo Fanizzi, Giuseppe Ciccarella

**Affiliations:** ^1^Institute of Nanotechnology, CNR Nanotec, Consiglio Nazionale Delle Ricerche, Via Monteroni, 73100 Lecce, Italy; ^2^Biological and Environmental Sciences Department, UdR INSTM of Lecce University of Salento, Via Monteroni, 73100 Lecce, Italy; ^3^DICATECh, Politecnico di Bari, Via Orabona, 70125 Bari, Italy

## Abstract

This study aimed to evaluate the therapeutic efficacy of low-intensity visible light responsive nanocolloids of a Pt-based drug using a 2D and three-dimensional (3D) *in vitro* cancer cell model. Biocompatible and biodegradable polymeric nanocolloids, obtained using the ultrasonication method coupled with Layer by Layer technology, were characterized in terms of size (100 ± 20 nm), physical stability, drug loading (78%), and photoactivation through spectroscopy studies. The *in vitro* biological effects were assessed in terms of efficacy, apoptosis induction, and DNA-Pt adducts formation. Biological experiments were performed both in dark and under visible light irradiation conditions, exploiting the complex photochemical properties. The light-stimuli responsive nanoformulation gave a significant enhancement in drug bioactivity. This allowed us to achieve satisfying results by using nanomolar drug concentration (50 nM), which was ineffective in darkness condition. Furthermore, our nanocolloids were validated in 3D *in vitro* spheroids using confocal microscopy and cytofluorimetric assay to compare their behavior on culture in 2D monolayers. The obtained results confirmed that these nanocolloids are promising tools for delivering Pt-based drugs.

## 1. Introduction

Nowadays, chemotherapy or radiotherapy is associated with insufficient therapeutic outcome and undesired side effects. Recently, important scientific advances have been achieved in the emerging era of personalized therapy, thanks to the integration of chemotherapy and targeted therapy with light irradiation [1]. Application of low-intensity light therapy has gained increasing attention for medical purpose in cancer treatment [2–6]. According to this method, the energy is transferred to living organisms inducing changes in biological processes [7–13]. The concept of low-intensity light therapy requires the use of photosensitive molecules, which respond to external light stimuli. In the last decade, delivery system-based phototherapies have been widely applied for curative and palliative treatment of cancers, [14, 15] including photodynamic therapy, [16] photoactivated chemotherapy, and [17] photothermal therapy, because it is possible to control the spatiotemporal activity of photoactivable molecules, enhancing the selectivity and limiting side effects [18].

A variety of light-responsive molecules could be used to obtain new chemotherapeutics contributing as a trigger for improved drug release and efficacy.

In recent years, there has been a growing interest in the synthesis of platinum(II) complex with natural substances [19, 20]. Among them, flavonoids and polyphenols are the most studied molecules. Curcumin belongs to polyphenol class and has been widely used in medicine because of its nontoxic nature and its antioxidant, anti-inflammatory, neuroprotective, and antiseptic activities [21].

As known, cisplatin (CisPt), which is a prototypical anticancer drug first described as chemical compound in 1844 [22–24], and its analogues have been widely used in cancer chemotherapy, but they are accompanied by severe adverse effects including hepatotoxicity, ototoxicity, nephrotoxicity, myelosuppression, and drug resistance [25, 26].

Photoexcitation of metal complexes has been extensively reported in the literature, with most examples dealing with their use in photoactivated chemotherapy for cancer treatment in the biomedical fields. Curcumin has been used as a photosensitizer [27], but its application in therapy is limited due to low solubility. Fortunately, curcumin metal complexes are usually more water soluble than free curcumin [17, 18, 28–30].

Stimuli responsive nanocarriers containing platinum were successfully tested in animal models, showing a remarkable reduction of side effects and decreased drug resistance [31–33].

**Scheme 1:**
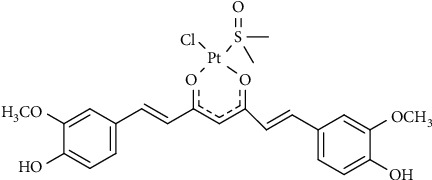
Chemical structure of PtCl (curc) (DMSO).

In the framework of the coordinative ability of curcumin towards Pt, recently, the complex PtCl(curc) (DMSO) (curc = deprotonated form of curcumin, DMSO = dimethyl sulfoxide) has been synthetized (scheme 1). In this complex, the natural ligand coordinates platinum via the oxygen atoms of the *β*-diketonate moiety, thus becoming more stable, because metal coordination prevented curcumin homopolymerization and photodegradation. In addition, the biological activity of PtCl(curc) (DMSO) in different cell lines resulted to be higher than the pristine Pt(II) complex and curcumin alone, suggesting a synergic anticancer effect between the Pt(II) center and the polyphenol ligand [19, 20].

Following our studies on curcumin-based platinum complex nanocolloids, herein, we report on the use of a nanotechnological approach to synthesize light-responsive polymeric nanocolloids of PtCl(curc) (DMSO). The presence of curcumin is useful for cellular imaging and allows us to obtain an improved biological efficacy, due to the synergic effect of platinum and curcumin on cancer cells, in both 2D and three-dimensional (3D) *in vitro* cancer models. Photoactivation was responsible for the biological action carried out by the nanoformulation.

A sonication-assisted layer by layer (LbL) assembly was the employed approach to obtain stable nanocolloids (Pt-Curc complex NCs) [34]. The polymeric nanocolloids were developed by the combination of two biopolymers, chitosan and pectin, which have received significant attention in recent years due to their abundance and natural availability. These biopolymers are used in many pharmacological and biomedical applications owing to their smart properties such as biocompatibility, biodegradability, and flexibility [35].

Photoactivated chemotherapy is the final purpose of this study. In this protocol, the molecules used as anticancer drugs should be endowed with negligible toxicity and biological activity when nonirradiated, while the visible irradiated form should exhibit strong biological activity [36].

During the past decades, traditional 2D cell cultures of immortalized cell lines have been extensively used for the evaluation of anticancer drug delivery systems. However, they have significant limitations related to the lack of many characteristics of the *in vivo* microenvironment conditions and complex cell-cell interactions. For this reason, in the last two decades, for an appropriate validation of pharmaceutical candidates, 3D-cell cultures, such as cellular spheroids, were introduced. These 3D models reflect the characteristics of solid tumors in terms of cell-cell and microenvironment interactions as well as molecular signaling profile and modulation of gene expression [37].

Thus, our work started with a deep physical-chemical characterization of polymeric nanocolloids. After reaching the desirable features of a drug delivery system, such as good aqueous solubility, colloidal stability, and controlled release pattern, we tested *in vitro* photoactivation of our nanoformulation. The biological experiments were carried out in a model cisplatin resistant cancer cell line, MCF7 (breast adenocarcinoma), both in 2D and 3D systems. The *in vitro* release behavior in terms of cytotoxicity and bioactivity of the nanoformulation was studied using UV-Vis spectroscopy, cytofluorimetric assay, and confocal investigation. Further analyses, including spheroids characterization and their 3D tumor size evaluation, were also carried out to better evaluate the toxicity of the nanoformulation.

## 2. Materials and Methods

### 2.1. Reagents and Instruments

Commercial products were purchased from Sigma-Aldrich. Glass bottom Petri dishes for confocal experiments were purchased from WillCo Dish. Deionized water was produced with a Millipore Elix® Deionizer with Pro-Gard® S2 ion exchange resin. Ultrasonication process was carried out with a VC 750 HV Sonics ultrasonicator.

### 2.2. Synthesis of [PtCl(curc) (DMSO)] and Preparation of Nanocolloids

For the synthesis of PtCl(curc) (DMSO) [20], a solution obtained by dissolving curcumin (0.0710 g, 0.1604 mmol) and KOH (9.0 mg, 0.1604 mmol) in ethanol (2.5 mL) was added dropwise to a solution of K[PtCl3(DMSO)] (0.0686 g, 0.164 mmol) in H_2_O (5.0 mL). The resulting dark mixture was stirred at room temperature for 36 h and then cooled at 277 K. After overnight at 277 K, a brown solid was removed by filtration, washed with ethanol and diethyl ether, and dried under high vacuum. Yield: 0.0786 g.

Nanocolloids were built up by sonication assisted layer-by-layer technique [38]. Biodegradable chitosan (CHI, polycation) and pectin (PEC, polyanion) were chosen for a biocompatible and biodegradable coating on drug nanoparticles. CHI solution was prepared at 0.10% in acid acetic 0.5 M; PEC aqueous solutions (0.5 mg·mL^−1^) were prepared in ultrapure water. The pH of the polymer solutions was adjusted to 5.0 by addition of acetic acid or NaOH/HCl, respectively. In the first step, 2.0 mg of PtCl(curc) (DMSO) [20] was added to 20 mL of a chitosan solution, stirred for 5 min, and then ultrasonicated for 20 min at 20% of power (150 Watt) in a water/ice bath, to make Pt-Curc/Chitosan nanocores. The following layer-by-layer self-assembly of pectin and chitosan was performed using LbL method. More in detail, the excess polycation was removed by three centrifugation/washing steps with deionized water. Thereafter, 5.0 mL of solution containing the polyanion was added, and the dispersion was continuously shaken for 20 min, followed again by three centrifugation/washing steps. This procedure was repeated 0.5, 2.5, or 4.5 times for the couple of polymers resulting in the deposition a polymeric shell with different thickness on the Pt-Curc nanocolloids (Pt-Curc/CHI; (CHI/PEC)_2.5_ (CHI/PEC)_4.5_).

### 2.3. Nanocolloids Characterization

TEM images were collected on JEOL JEM 1400 with a LaB6 source at 80 kV. The zeta potential and the hydrodynamic diameter of the NCs were measured with Malvern Zetasizer Nano ZS (Dynamic Light Scattering analysis).

The UV–Vis absorption and emission spectra were obtained on a Varian-Cary 500 spectrophotometer and a Varian Cary Eclipse spectrofluorometer, respectively. Absorption and fluorescence spectra were measured in deionized water, with a final concentration of 50 *µ*M. The quartz cuvettes used were of 1.0 cm path length.

### 2.4. Cisplatin-Loading and Quantiﬁcation by ICP-OES

Drug loadings were quantified by inductively coupled plasma optical emission spectrometry (ICP-OES). The experimental setup provides the construction of a calibration line, using 4 points. Loading percentage is defined as the number of platinum ppt in the solution after loading divided by the number of platinum ppt in the solution before loading. ICP experiments were performed with an ICP-OES Thermo Scientific instrument.

### 2.5. Visible Light Irradiation

The samples were put in a flat-bottom 6-well microplate above the xenon lamp (Spectral Products mod.1118263) for 1 h (at a distance of 3.5 cm). During irradiation, the temperature was kept at 23 ± 2°C. Native samples to be irradiated were obtained from the same stock. After irradiation, the samples were kept in the CO_2_ incubator for 20 h at 37°C before further analysis.

### 2.6. Release Kinetics

The release kinetics was evaluated in phosphate buffer at physiological and acidic pH. Pt-Curc NCs were constantly stirred to increase the release rate and to establish the equilibrium conditions. Separate tubes were used for each time. At each time point, 500 *µ*L of release media was replaced with the same volume of fresh release media. At selected time intervals, NCs were separated by centrifugation. The supernatant was collected, and the platin content in the supernatant was determined by ICP-OES analysis.

### 2.7. Incubation and Staining of Living Cells

MCF7 cancer cell line was maintained in Dulbecco's modified Eagle's medium (DMEM) supplemented with fetal bovine serum (FBS) (10%), penicillin (100 U·mL^−1^ culture medium), streptomycin (100 mg·mL^−1^ culture medium), and glutamine (5%). Cells were grown in a humidified incubator at 37°C, 5% CO_2_, and 95% relative humidity. Cell line was serum-starved for 24 h before any test. For staining experiments, MCF7 (5 × 10^4^) cells were seeded onto a 35 mm glass bottom Petri dish and incubated in complete media for 24 h. Then, the cells were incubated with the curcumin or Pt-Curc complex at a concentration of 50 *µ*M for 30 min or 2 hours in the dark in a humidified incubator at 37°C, 5% CO_2_, and 95% relative humidity. After rinsing with phosphate buffered saline (PBS) twice, cells were stained with Hoechst and imaged by confocal microscopy, immediately adding complete media without red phenol.

### 2.8. Confocal Imaging of Living Cells

Biological imaging tests were carried out with a Zeiss LSM700 confocal microscope (Zeiss, Germany) equipped with a Zeiss Axio Observer Z1 inverted microscope using a 63X objective with a 1.46 numerical aperture oil immersion lens for imaging. Laser beams with 405 nm and 488 nm excitation wavelengths were used for Hoechst and curcumin or Pt-Cur complex NCs imaging, respectively.

### 2.9. Generation of 3D Spheroids Using Low-Adhesion Agarose Plate

A solution of 1% (wt/vol) of agarose was prepared mixing 0.5 g of molecular biology–grade agarose in 50 mL of PBS. The mixture was heated in a microwave for 1–2 min (with pauses to prevent boiling) until the agarose was completely dissolved. 50 *μ*L of agarose solution was plated into each well of a 96-well plate while it was still hot, using sterile pipette tips. After gelation at room temperature, MCF7 cell line was seeded to obtain the spheroids as follows.

The human breast cancer cell line, MCF7, cultured as 2D monolayers in DMEM medium, was trypsinized, counted, and plated at a density of 4.0 × 10^4^ cells/mL in complete growth medium. Finally, the multiwell was transferred into the incubator. After 24 h incubation, tumor spheroids could be observed in each well. The medium was changed every day.

The morphological characteristics of the spheroids, including their diameter and shape, were determined by optical analysis using EVOS XL Cell Imaging System microscope (Thermo Fisher, Waltham, MA, USA). The progressively developing spheroids were observed at 24 h intervals. The mean diameter of the 3D structures was calculated by using ImageJ Software (1.48 v).

### 2.10. Cytotoxicity Determination by MTT Assay

MCF7 cancer cell line was used in the general cytotoxicity test. The MTT method was used to measure the activity of living cells via mitochondrial dehydrogenase activity. The key component was 3-[4,5-dimethylthiazol-2-yl]-2,5-diphenyltetrazolium bromide or MTT. Mitochondrial dehydrogenases of viable cells cleave the tetrazolium ring, yielding purple MTT formazan crystals, which are insoluble in aqueous solution. The MTT method is most effective when cultures are prepared in multiwell plates. Cells (3,5 × 10^4^ cells per mL) were added to 24-well culture plates at 0.5 mL per well, serum-starved for 24 h, and incubated at 37°C, 5% CO_2_, and 95% relative humidity for 24 hours with curcumin, Cisplatin, or Pt-Curc complex NCs. The control was a complete culture medium. After 24 h of incubation, cultures were removed from the incubator, and MTT solution (10% of the culture volume) was aseptically added. Cultures were returned to the incubator and incubated for 3 hours. After the incubation period, the cultures were removed from the incubator, and the resulting MTT formazan crystals were dissolved in DMSO (using the same volume of the culture). The plates were ready within 15 min after adding DMSO. After the incubation time, pipetting up and down was required to completely dissolve the MTT formazan crystals. Absorbance at a wavelength of 570 nm was measured using a spectrophotometer. Results were expressed as mean ± SD of three separate trials.

### 2.11. Flow Cytometric Analysis of Apoptosis

The Annexin V-fluorescein isothiocyanate (FITC) kit (Thermo Fisher Scientific, Inc.) was used to determine cellular apoptosis. After exposure to curcumin, Cisplatin, or Pt-Curc NCs at the IC_50_ concentration for 24 h in 37°C, MCF7 cells were collected, washed twice with PBS, and subjected to centrifugation at 1200 × *g* for 5 min at room temperature. Subsequently, the cell pellet was resuspended and treated with Annexin V-FITC and propidium iodide (PI) solutions. After incubating for 15 min at room temperature in the dark, additional Annexin V binding buffer (10 mM Hepes, 140 mM NaCl, 2.5 mM CaCl_2_ in ddH_2_O) was added to each tube, and the cells were analyzed.

To analyze the apoptosis in 3D spheroids, 5-day-old spheroids were chosen and exposed to curcumin, Cisplatin, or Pt-Curc complex NCs at the IC_50_ concentration, for 24 h at 37°C. After the incubation period, all spheroids were rinsed with 10 mL of PBS and were collected in a 15 mL tube. After centrifugation (5 min, 400 *g*), spheroids were trypsinized to obtain a single cell suspension by gentle pipetting. Subsequently, the cell pellet was resuspended and treated with Annexin V-FITC and propidium iodide (PI) solutions.

Flow cytometry was performed on a flow cytometer (BD Biosciences, San Jose, CA, USA) equipped with FlowJo software.

### 2.12. Cellular Uptake for Pt Estimation

To measure the cellular platinum uptake, about 10^6^ MCF7 cells were seeded in 75 mm tissue culture dishes and treated with Cisplatin (35 *μ*M), Cisplatin and curcumin (35 *μ*M and 10 *μ*M), and Pt-Curc NCs (50 nm) for 24 h in the dark. After incubation, we proceeded with DNA extraction using PROMEGA kit (Wizard® Genomic DNA Purification Kit). The cells were harvested in medium and then washed twice with PBS and suspended in Nuclei Lysis Solution pipetting to lyse the cells. Then, few µL of RNase Solution was added to the nuclear lysate. After incubation time at 37°C, to the room temperature, the Protein Precipitation Solution was added. After centrifugation, the precipitated protein formed a tight white pellet. Carefully, we removed the supernatant containing the DNA and transferred it to a clean microcentrifuge tube containing 600 *µ*L of room temperature isopropanol. After centrifugation, 70% ethanol was added. The DNA pellet was collected after centrifugation, and it was incubated with DNA Rehydration Solution at 65°C for 1 h. DNA was then quantified using a NanoDrop Spectrophotometer. The amount of platinum on the DNA was determined by using ICP-MS. The concentration of platinum was expressed as the percentage of platinum content in comparison with the platinum estimated in the fed solution.

The samples were analyzed using ICP-OES for platinum content along with the standards (0.01–1 ppm, and a blank sample fitted in a linear plot with a correlation of 0.99). All experiments were performed in duplicates along with untreated controls.

To estimate the Pt content in 3D spheroids, after the exposure to Cisplatin or Pt-Curc NCs at the same concentrations described above, for 24 h at 37°C, all spheroids were rinsed with 10 mL of PBS and were collected in a 15 mL tube. After centrifugation (5 min, 400 *g*), spheroids were trypsinized to obtain a single cell solution and then processed for DNA extraction. All experiments were done with compound pretreated MCF7 cells with light exposure of 60 min (400–700 nm, 5 J·cm^−2^).

### 2.13. Statistical Analysis

Statistical differences between control and drug-treated cells were determined by one-way ANOVA (Sidak). *P* values <0.05 were considered statistically significant. Data were analyzed using the Stata 8.2/SE package (StataCorp LP).

## 3. Results and Discussion

### 3.1. Physical Chemical Characterization

Ultrasonication assisted LbL assembly process was used to obtain stable nanocores of Pt-Curc complex starting from drug microcrystals. In this method, the complex PtCl(curc) (DMSO) was added to a chitosan solution (0.10%) prepared in acetic acid 0.5 M. The ultrasonication duration time was fixed to 20 min because beyond this time the particle size did not decrease further. During sonication, the simultaneous deposition of chitosan layer over the drug nanocore prevented the aggregation and stabilized the mixture, thanks to the strong positive charge derived from this polymer. LbL assembly was performed by sequentially adsorbing oppositely charged polymers: pectin and chitosan.

During LbL process, the value of zeta potential was measured after the deposition of each layer. The charge switched from the positive layer of chitosan to the negative layer of pectin. The values of the zeta potential were always around ±25–30 mV. The colloidal stability is one of the main features of a nanoformulation since it is important to preserve the physicochemical properties. The ultrasonication protocol, combined with LbL technology, is carried out carefully controlling the thickness of the multilayer shell and its chemical composition. For this reason, Pt-Curc NCs with different polymeric shell thickness were prepared using either chitosan alone, or bilayers of chitosan and pectin (CHI/PEC)_2.5_ or four bilayers of chitosan and pectin (CHI/PEC)_4.5_. Stability studies of Pt-Curc complex NCs were performed in deionized H_2_O at room temperature, measuring the zeta potential and the size every day over 2 weeks (Figure S1). The NCs stability was found dependent on the synthetic procedure. NCs coated with only one layer of chitosan showed, after one week, a decrease in zeta potential value, accompanied by an increase in size dimension, probably due to the aggregation. The (CHI/PEC)_2.5_ bilayers coating provided the best colloidal stability, as indicated by the unvaried size and zeta potential over 2 weeks (Figure S1). The increment of bilayers, (CHI/PEC)_4.5_, led to a fast NC aggregation. These results are in accordance with literature, suggesting that the colloidal stability is influenced by the number of bilayers deposed around the drug nanocore [34, 39]. Increasing the number of bilayers, the polymeric system probably tends to compact itself and nanocolloids and tends to aggregate, probably also for macromolecular structure, concentration, and charge density of polymers. It should be recalled that the growth mechanism of multilayer is attributed to the dissociation of ionizable functional groups, secondary interactions, or diffusion of polymers through the formed shell [30, 40–42].

The best nanoformulation, Pt-Curc NCs (CHI/PEC)_2.5_, was used to determine the loading efficiency of drug inside the polymeric shell. ICP-OES measurements estimated an efficiency of around 78% (Table 1).

**Figure 1:**
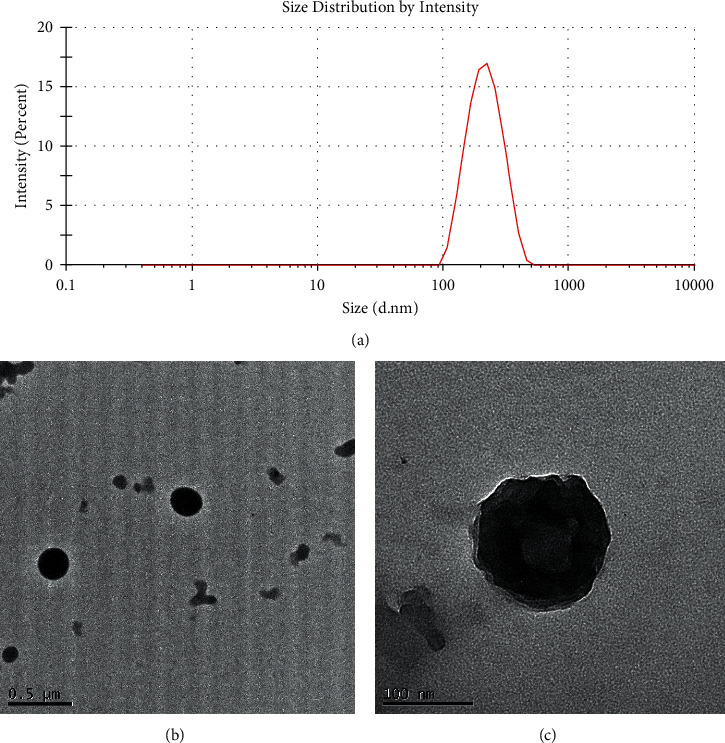
Intensity profile of Pt-Curc NCs measured by DLS (a) and TEM images (b and c) of Pt-Curc NCs obtained after 20 min of ultrasonication in chitosan solution. Scale bars are reported in each image.

The monodispersity of these nanocolloids (NCs) was confirmed using the dynamic light scattering (DLS) technique. The shape of the NCs was homogeneous, with no observable aggregates; nanocores with a zeta potential of about 28.81 ± 1.53 mV and diameter of 120 nm were obtained (Figure 1(a)). As shown in TEM images (Figures 1(b) and 1(c)), NCs exhibit a spherical shape with an average diameter of about 100 ± 20 nm. These results are summarized in Table 1.

**Figure 2:**
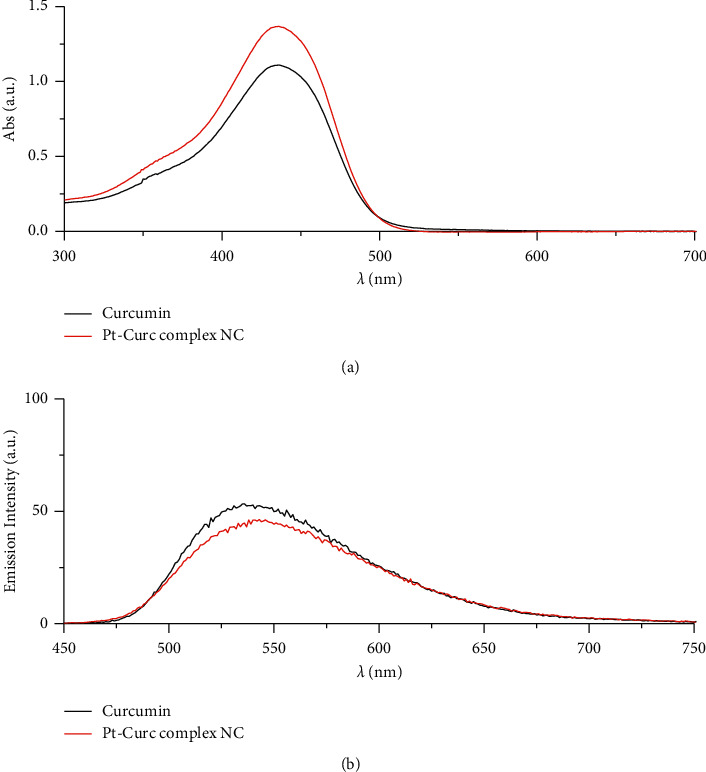
(a) UV/Vis spectra of curcumin (black line) and Pt-Curc NCs (red line) in PBS (pH = 7.2). (b) Emission spectra of curcumin (black line) and Pt-Curc NCs (red line) in PBS (excitation wavelength = 430 nm).

In this study, the Pt-Curc NCs were also subjected to photostability studies by monitoring UV/Vis and PL spectral changes with time (Figure 2). Curcumin ligand alone was observed to show the stability in the buffer medium (PBS—phosphate-buffered saline, pH = 7.2), as reference. It was prepared in ethanol, and the tested solution (50 *µ*M) was prepared in PBS. The Pt-Curc complex NCs were prepared at the same concentration in PBS.

The absorbance and the emission intensity were monitored for two weeks storing the samples in the dark at 4°C. The absorption spectra of Pt-Curc complex NCs displayed a broad intense absorption band at 450 nm, like curcumin. The emission spectra of Pt-Curc NCs and curcumin resulted also to be similar, showing a characteristic band at 530 nm (*λ*_ex_ = 430 nm). Pt-Curc NCs had a slightly lower emission intensity probably due to the presence of platinum. This behavior is already reported in the literature for other Pt-complexes; however, to the best of our knowledge, the same measurements related to nanocolloids have never been recorded yet [43].

**Figure 3:**
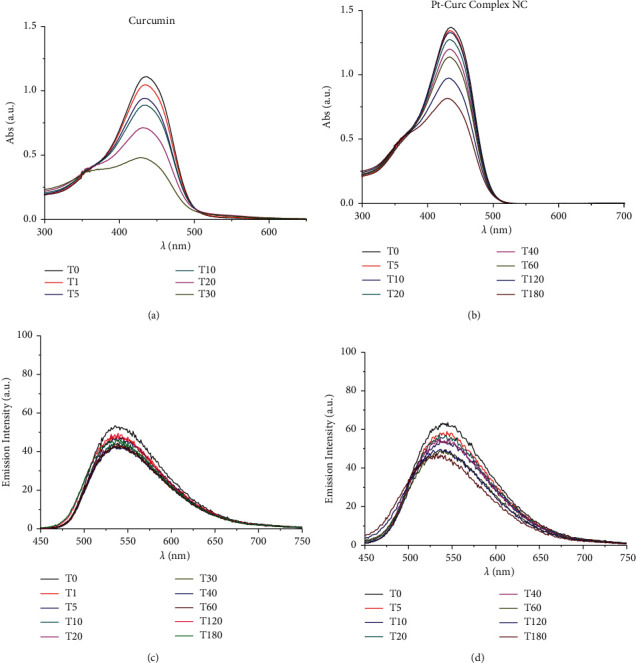
UV/Vis absorption spectra of curcumin (a) and Pt-Curc NCs (b) in PBS (pH = 7.2). Emission spectra of curcumin (c) and Pt-Curc NCs (d) in PBS (excitation wavelength = 430 nm).

Furthermore, changes in UV/Vis and PL spectra were monitored to study the photoexcitation and the stability under light irradiation. Samples were irradiated with visible light (400–700 nm, 2.5 J·cm^−2^), and the absorbance and fluorescence emission were recorded every 5 minutes for 3 hours. Results are reported in Figure 3. As expected, at the beginning, curcumin displayed an intense absorption band at 450 nm that rapidly degraded upon irradiation: just after 30 min, the peak intensity was halved. Pt-Curc NCs, at the same concentration, showed a different absorption spectrum that did not reach half of the absorption even after 3 hours of irradiation (Figure 3(b)). The emission intensity of free curcumin and Pt-Curc NCs was almost comparable.

UV/Vis and PL investigations suggest that Pt-Curc NCs are stable in darkness and that their intensity remained unchanged; however, the light is necessary for their activation.

In the literature, similar complexes, although not in nanoformulation form, showed different behavior. The photo exposure led to a gradual enhancement of emission intensity at 530 nm that was accompanied with a gradual decrement of absorbance, probably due to the detachment of curcumin [44–47].

### 3.2. Loading Efficiency and Release

The loading efficiency and the release rate were measured by ICP-OES. As reported above, quantitative measurements revealed a loading efficiency of about 78% for Pt-Curc NCs. Different polymeric materials have been used as drug delivery vehicles. Each type of polymer (natural, semisynthetic, and synthetic) has its own specific features, and the physicochemical limitations could tune the release rate and the targeting of the active molecules to a specific site of action. In this regard, polymer blending has been considered as an attractive approach to fabricate novel and unique drug delivery systems with modified physical and/or chemical characteristics. Drug release from these polymeric systems can be tuned through the changes in temperature and pH of the environment, and physiochemical properties of the target organs. It is important to consider that possible molecular interactions between polymers and drug molecules can significantly affect the drug release profile from these blended polymeric carriers. As shown in Figure S2, the release kinetics are affected by light exposure and pH condition. The *in vitro* release profile has been studied at pH 7.5 and 5.4, monitoring Pt-Curc NCs suspension at 1.0 mg/mL from 1 min to 72 h. Chitosan is sensitive to acid pH [48], since the protonation of the amino group occurs when the pH is below its isoelectric point (6.3). For this reason, the kinetics were faster at pH 5.4. In particular, at neutral pH (Figure S2(a)), in dark condition, the sample reached the plateau of about 40% release after 48 h. Visible light irradiation led to a discrete release of platinum from NC. The release reached the 80% after 72 h.

In acid condition (Figure S2(b)), the release profiles (in darkness and visible-light irradiation) are almost the same because the low pH favors the chitosan degradation also in dark condition, hiding the effect of light exposure.

### 3.3. Biological Activity

#### 3.3.1. Cell Viability Studies

Drug delivery systems play an important role in cancer treatment as they can directly target cancerous cells increasing drug concentration in the tumor environment [49, 50]. Several studies demonstrated that nanocarriers increased the cytotoxicity effects on the cancerous cells [51, 52].

MTT assay was performed on human breast cancer cell line (MCF7) in the dark and in light conditions, in order to study the cytotoxicity of nanocolloids [53]. At first, cells were incubated with different concentrations of curcumin, cisplatin, and both drugs, to evaluate the synergic effect of these two drugs (cisplatin 35 *µ*M and curcumin 10 *µ*M) [54], and Pt-Curc NCs in darkness to obtain the IC_50_ values. The cytotoxicity data was shown in Figure S5. The estimated IC_50_ values are 75 *µ*M for curcumin, 35 *µ*M for cisplatin, and 50 *µ*M and Pt-Curc NCs. In the case of synergic treatment, the presence of polyphenol (10 *µ*M) sensitizes the cells to the biological action of cisplatin (Figure S3).

These values, obtained in dark condition, were compared with the IC_50_ estimated in our previous work [20] and suggested that the nanocolloids improved the bioavailability of platinum complex showing a cytotoxic effect 20% higher.

These values were used for photo exposure experiments. More in detail, the experimental setup provides the incubation of MCF7 cells with both curcumin (75 *µ*M) and Pt-Curc NCs (50 *µ*M) for 4 h in the dark, and then, the cells were subjected to irradiation with visible light for 1 h (400–700 nm, 2.5 J·cm^−2^). The photoexcitation of cells incubated with Pt-Curc NCs led to an abrupt decrease in cell viability (data not shown), confirming that nanocolloids, after photoirradiation, can make the Pt-complex immediately available to cells.

However, when similar photo exposure experiments were carried out by using PtCl(curc) (DMSO), as molecule as it is, after irradiation, a lowering of cell viability was observed, with a slight reduction of IC_50_ value [20].

**Figure 4:**
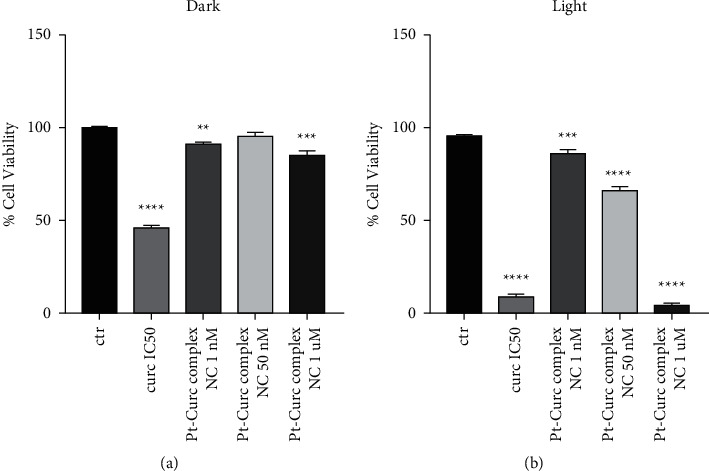
Cytotoxicity effect of curcumin and Pt-Curc NCs for 24 h in the dark against MCF7 cell lines (a). Cytotoxicity effect of curcumin and Pt-Curc NCs against MCF cell lines exposed to visible light (b) for 1 h (400–700 nm, 2.5 J·cm^−2^). Values represent mean + SD and were obtained from three independent experiments. Statistically significant value *p* < 0.01 (^*∗∗*^), very statistically significant *p* < 0.001 (^*∗∗∗*^), and extremely statistically significant *p* < 0.0001 (^*∗∗∗∗*^).

Since photoactivation increases the toxicity of our nanoformulation, it was necessary to investigate a nanomolar range of concentration (1.0 nM and 1000 nM). The results reported in Figure 4 indicate that photoirradiation did not affect cell health (black histogram). The IC_50_ of curcumin (75 *µ*M) caused a substantial decrease in cell viability under light irradiation (10, 5%). In the case of Pt-Curc NCs, the nanomolar concentrations, which in darkness gave obviously no effect on cell viability, under photoactivation, surprisingly, led to an increase in cytotoxicity. In particular, Pt-Curc complex NCs at concentration of 1.0 nM affected on cell viability decreasing the value by about 20%; at 50 nM, the cell viability decreased significantly at 50%.

**Figure 5:**
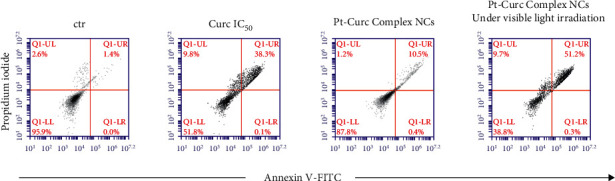
Apoptosis of MCF7 cells mediated by curcumin, Pt-Curc NCs without irradiation, and Pt-Curc NCs under visible light irradiation. Apoptosis was measured by flow cytometry after PI/Annexin V-FITC staining. Q1-UL, PI+ (cells undergoing necrosis); Q1-UR, Annexin V-FITC + PI+ (cells in the late period of apoptosis and undergoing secondary necrosis); Q1-LR, Annexin V-FITC + PI− (cells in the early period of apoptosis); Q1-LL, Annexin V-FITC− PI− (living cells).

To gain insight into biological effect of Pt-Curc NCs, the nanoformulation was tested on MCF7 cells in order to check the induction of apoptosis by using Annexin V/PI double staining, in a cytofluorimetric assay. Cells were treated with curcumin at IC_50_ (75 *µ*M), Pt-Curc NCs (50 nM) without irradiation and Pt-Curc NCs (50 nM) under visible light irradiation. Results shown in Figure 5 demonstrate that, in dark condition, curcumin led to a reduction of cell viability of 50%; whereas the Pt-Curc NCs showed a negligible cytotoxicity (10%). Instead, under visible light irradiation, the apoptotic rate significantly increased when MCF7 was treated with Pt-Curc NCs at the same nominal concentration (50 nM).

#### 3.3.2. Cytotoxicity toward 3D Spheroids

The cytotoxicity studies were conducted on 3D spheroids as a tumor model to evaluate the therapeutic efficacy of Pt-Curc complex NCs. Spheroids, which were generated in a highly reproducible manner, represent one of the simplest methodologies used for drug screening in cancer research, because they mimic the *in vivo* environment and are more physiologically relevant system than 2D monolayers [55].

At first, the growth of spheroids was monitored using optical microscope (Figure S4). The morphological changes during spheroid formation involved different stages [56]. At the beginning, day 1, thanks to the hydrophobicity of the substrate, single cells suspension spontaneously self-assembled to form cell aggregates. After 48–72 h, cell aggregates and the spheroids become more compact due to cellular reorganization and extracellular matrix (ECM) secretion. After 3–5 days, the spheroids revealed a smooth and continuous surface, and the morphology did not change significantly. For this reason, all tests were performed at day 5 after seeding.

To investigate the biological efficacy of Pt-Curc NCs, 3D spheroids of MCF7 tumor cells with an average diameter of ∼300−400 *µ*m were exposed for 24 h to either free Curcumin at IC_50_ concentration value (75 *µ*m), Cisplatin at IC_50_ concentration value (35 *µ*M), and Pt-Curc NCs (50 nM).

A morphological analysis and cytotoxic evaluation were carried out. After 24 h of incubation with all tested compounds, cells exhibited clear signs of cytotoxicity: spheroids lost the cohesion, and the edges became irregular. This finding was particularly prominent in MCF7 spheroids after the treatment with Pt-Curc NCs and the exposure to light irradiation (Figure S4).

**Figure 6:**
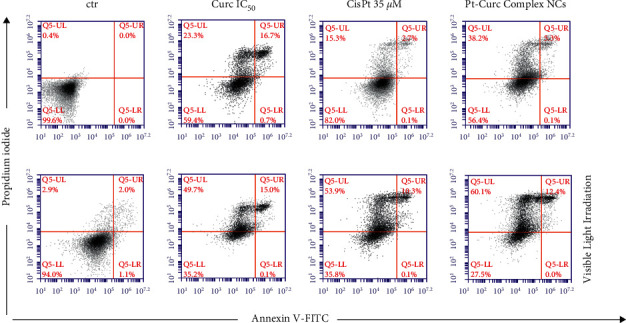
Apoptosis of spheroids of MCF7 cells mediated by curcumin, Cisplatin, and Pt-Curc NCs after 24 h in the dark (top series) or under visible light irradiation (bottom series). Apoptosis was measured by flow cytometry after PI/Annexin V-FITC staining. Q1-UL, PI+ (cells undergoing necrosis); Q1-UR, Annexin V-FITC + PI+ (cells in the late period of apoptosis and undergoing secondary necrosis); Q1-LR, Annexin V-FITC + PI− (cells in the early period of apoptosis); Q1-LL, Annexin V-FITC− PI−(living cells).

The association of the altered morphology and the viability of the spheroids was evaluated via flow cytometry using Annexin-PI test (Figure 6). The results obtained in dark condition indicated that, after 24 h of treatment, the cytotoxicity induced by curcumin was around 40%, while, in the case of Cisplatin, the value was around 20%. Instead, the treatment with Pt-Curc Complex NCs led to a cytotoxicity of about 50%.

This difference in cytotoxicity, even after 24 h of incubation, can be explained admitting that the nanocolloids have been actively taken up by tumor 3D spheroids and readily penetrate them controlling the release of curcumin and platinum. This behavior can be, also, justified by high sensitivity of the cells in S phase of the cell cycle. The cytotoxic mode of action of metal drug is mediated by its interaction with DNA and leading up in the activation of apoptosis [57, 58].

This effect is clearer after visible light irradiation. Curcumin and cisplatin gain a significant cytotoxicity, reaching a value of about 65% in both cases; but the effect of our nanoformulation is more evident.

In fact, the treatment with Pt-Curc NCs under visible light irradiation led to a decrease in cell viability up to 72.5%. This demonstrates that the development of a water soluble nanocarrier containing curcumin and platinum increases the penetration inside tumor spheroids resulting in higher cytotoxic effects.

#### 3.3.3. Cell Uptake Studies in 2D and 3D Spheroids

**Figure 7:**
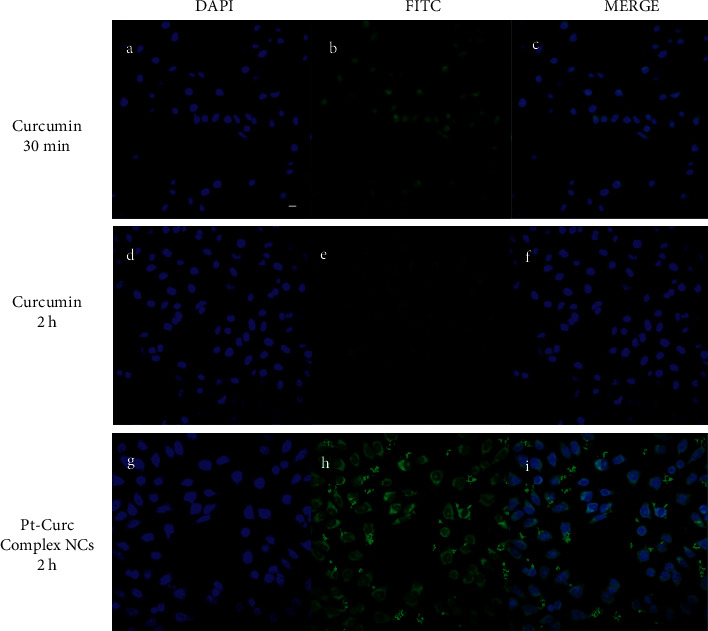
Confocal images in MCF 7 cells treated with 50 *μ*M of curcumin and 50 *μ*M of Pt-Curc NCs in the dark for 30 and 2 hours. Scale bar = 5 *μ*m.

Confocal microscopy experiments were carried out to study the cellular localization of Pt-Curc NCs. Exploiting curcumin fluorescence, it is possible to track nanocolloids localization inside cells without using other fluorescent probes. We imaged the cells after two time points of incubation, 30 min, and 2 h, with curcumin alone and Pt-Curc NCs.  Figure 7 shows the relevant confocal micrographs; in blue are the stained nuclei of MCF7 cells and in green is the fluorescence associated to curcumin.

The internalization was very fast in MCF7 cells; confocal microscopy investigation clearly confirmed that curcumin efficiently and rapidly entered cells and showed its main cytoplasmic localization within 30 min (Figures 7(a)–7(c)). As reported in the literature, curcumin degraded rapidly [59, 60], and indeed no fluorescence appears after 2 h of incubation in darkness (Figures 7(d)–7(f)). Conversely, in the case of Pt-Curc NCs, the curcumin is firmly bonded to the platinum center, and we observed intense fluorescence even after two hours of incubation (Figures 7(g)–7(i)) (z-stack micrographs were reported in Figure S5). This observation is in line with results obtained in *in vitro* stability tests.

The targeting of solid tumor tissue is blocked by the restricted interstitial transport, which reduces chemotherapeutic drug diffusion *in vivo* [61]. For this reason, in the present study, to better mimic the extracellular microenvironment, the internalization process of our nanoformulation was also investigated in MCF7 3D spheroids [62–64].

**Figure 8:**
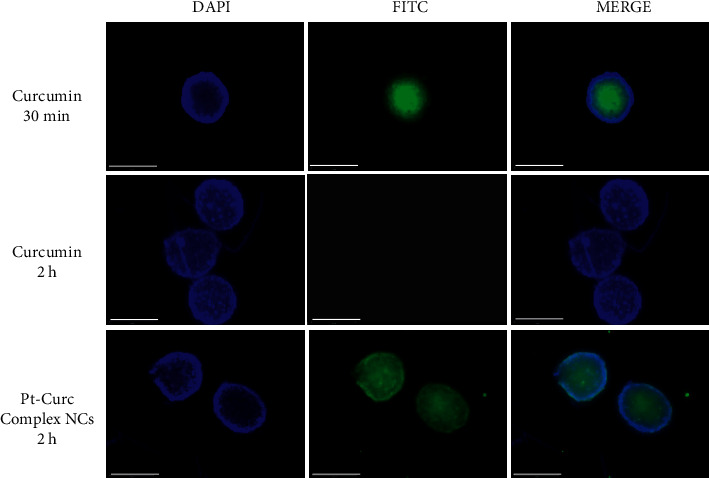
Representative images of MCF7 spheroid cross-sections incubated with Curcumin 50 *μ*M for 30 min and 2 h (first and second lines) and Pt-Curc-complex NCs 50 *μ*M for 2 h (third line). Scale bars, 300 *μ*m.

Figure 8 displays representative spheroid cross-sections observed by confocal microscopy (see videos in SI). The images show that Pt-Curc NCs induced a strong green fluorescence, which indicates the uptake of nanocolloids by 3D spheroids. The fluorescence signal persists for long time. The behavior of curcumin alone was the same evidenced in 2D monolayer; that is, it was degraded within 10 h.

#### 3.3.4. Pt Estimation

**Figure 9:**
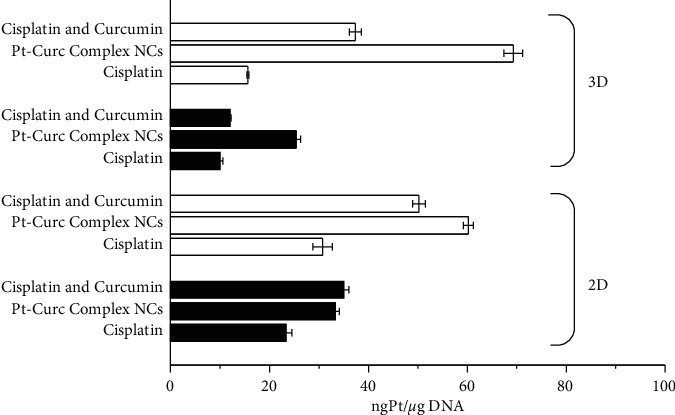
DNA platination of MCF7 cells in 2D monolayer and 3D spheroids in dark (black histograms) and under visible light irradiation (white histograms). The cells were incubated with Cisplatin, Cisplatin and Curcumin, and Pt-Curc complex NCs. The total incubation period was 24 h either in the dark or exposed to light (400–700 nm, 2.5 J·cm^−2^) for 1 h after 4 h in the dark. The concentration tested was the IC_50_ for all conditions. Values represent mean ± SD and were obtained from three independent experiments.

The platinum(II) content was estimated by ICP-OES . MCF7 cells in 2D monolayer and 3D spheroids were treated at the IC_50_ concentrations with Cisplatin (35 *µ*M), Cisplatin and curcumin (35 *µ*M and 10 *µ*M, respectively), and Pt-Curc complex NCs (50 nM). The experiments were carried out both in dark and under light irradiation. The Pt content was evaluated in the nuclear fraction (Figure 9).

Pt estimation by ICP-OES confirmed that, in darkness condition, a scarce quantity of platinum reaches the nucleus, and also in the case of Pt-Curc complex NCs. In the case of 3D spheroids, it is evident that the nanoformulation readily penetrates tumor cells aggregate. Results indicate that the photo exposure encourages the Pt(II) migration from cytosol to nucleus, because the Pt content in this fraction is abundant. This datum indicates the successful photoactivation of the complex. Pt-Curc complex NCs showed a higher cellular accumulation in 3D spheroids in comparison to 2D monolayer, suggesting that the solubility of the nanoformulation is essential for a biological efficacy. These data confirm that light exposure is an effective way to improve drug efficacy minimizing side effects.

## 4. Conclusion

In the present study, a light stimuli responsive polymeric delivery system for Pt-Curc complex was prepared and deeply characterized.

We developed a water soluble and visible light-induced apoptosis activatable nanoformulation of a metal curcumin complex with an average size of 120 nm. Unlike conventional photo therapy using high-energy light, in the present work, low-energy irradiation was enough to induce apoptosis in cancer cells.

All biological experiments, carried out both in 2D and in 3D systems, demonstrated the therapeutic efficiency of Pt-Curc NCs, in terms of cell internalization and biological effect.

The polymeric drug delivery system showed a behavior superior to other nanocarriers reported in the literature, which have shown a fast exocytosis and are associated to a lower therapeutic performance [65]. Using light irradiation, it is possible a fine control of Pt(II) release from Pt-Curc NCs without a slow diffusion that normally occurs in other drug delivery systems. A fast cellular internalization, especially in 3D tumor spheroids, is ensured by the employment of cationic polymers [66], chitosan, on the external surface.

Exploiting the photoactivation, it is possible to use nanomolar concentration of Pt-Curc NCs obtaining a material biologically inactive in dark conditions and very effective under light irradiation. This is an advantage because it is possible a fine control during administration. This great cytotoxic effect has been explained by NCs stability inside cancer cells and high targeting capability.

Both physical characterization and biological experiments suggest that the nanoformulation of Pt-Curc NCs is very stable inside cancer cells because the fluorescence persists for a long time, whereas the curcumin alone degraded rapidly. The estimation of DNA-Pt adducts showed that a nuclear targeting is achieved by the active form of molecular Pt(II) complex NCs following light-activation.

Thus, we argue that the Pt-Curc NCs activated via light-induced apoptosis can overcome many limitations of the current light cancer therapy such as low efficacy, insolubility of photosensitizers, poor tissue penetration, and rapid oxygen depletion [67].

## Figures and Tables

**Table 1:** Size, size distribution, zeta potential, and encapsulation loading efficiency of Pt-Curc NCs.

Formulation	Size (nm)	Size distribution (nm)	Zeta potential (mV)	Encapsulation efficiency (%)
Pt-Curc NCs	100 ± 20	0.048 ± 0.003	28.81	78

## Data Availability

The authors confirm that the data supporting the findings of this study are available within the article and its supplementary materials.

## Supplementary Materials

Supplementary Materials The supplementary file contains S1 figure with Nanocolloids size and Zeta potential measurements; S2 figure with Release kinetics; S3 figure with Cytotoxicity test performed on MCF 7 cell lines; S4 figure with Optical representative images of spheroids of MCF 7 cell lines; S5 figure with Confocal Z-stack micrographs of MCF7 treated with Curcumin (50 *μ*M) and Pt-Curc NCs (50 *μ*M) in the dark for 2 hours.

## References

[1] Marur S., Forastiere A. A. (2010). Challenges of integrating chemotherapy and targeted therapy with radiation in locally advanced head and neck squamous cell cancer. *Current Opinion in Oncology*.

[2] Smith K. C. (2005). Laser (and LED) therapy is phototherapy. *Photomed Laser Surg*.

[3] Oakley M. S., Gerald N., Anantharaman V. (2013). Radiation-induced cellular and molecular alterations in asexual intraerythrocytic plasmodium falciparum. *The Journal of Infectious Diseases*.

[4] Pinheiro A. L. B., Nascimento S. C., de Barros Vieira A. L. (2002). Effects of low-level laser therapy on malignant cells: in vitro study. *Journal of Clinical Laser Medicine and Surgery*.

[5] Morton C. A. (2004). Photodynamic therapy for nonmelanoma skin cancer—and more?. *Archives of Dermatology*.

[6] Schindl A., Schindl M., Pernerstorfer-Schön H., Mossbacher U., Schindl L. (2000). Low intensity laser irradiation in the treatment of recalcitrant radiation ulcers in patients with breast cancer – long-term results of 3 cases. *Photodermatology, Photoimmunology & Photomedicine*.

[7] Izzotti A., Calin G. A., Steele V. E., Croce C. M., De Flora S. (2009). Relationships of MicroRNA expression in mouse lung with age and exposure to cigarette smoke and light. *The FASEB Journal*.

[8] Vinck E. M., Cagnie B. J., Cornelissen M. J., Declercq H. A., Cambier D. C. (2003). Increased fibroblast proliferation induced by light emitting diode and low power laser irradiation. *Lasers in Medical Science*.

[9] Cosic I., Vojisavljevic V., Pavlovic M. (1989). The relationship of the resonant recognition model to effects of low-intensity light on cell growth. *International Journal of Radiation Biology*.

[10] Cosic I. (1994). Macromolecular bioactivity: is it resonant interaction between macromolecules?-theory and applications. *IEEE Transactions on Biomedical Engineering*.

[11] Bruzell E. M., Morisbak E., Tønnesen H. H. (2005). Studies on curcumin and curcuminoids. XXIX. Photoinduced cytotoxicity of curcumin in selected aqueous preparations. *Photochemical and Photobiological Sciences*.

[12] Lipovsky A., Oron U., Gedanken A., Lubart R. (2013). Low-level visible light (LLVL) irradiation promotes proliferation of mesenchymal stem cells. *Lasers in Medical Science*.

[13] Hopkins S. L., Siewert B., Askes S. H. C. (2016). An in vitro cell irradiation protocol for testing photopharmaceuticals and the effect of blue, green, and red light on human cancer cell lines. *Photochemical and Photobiological Sciences*.

[14] Luo D., Carter K. A., Molins E. A. G. (2019). Pharmacokinetics and pharmacodynamics of liposomal chemophototherapy with short drug-light intervals. *Journal of Controlled Release*.

[15] Li M., Nguyen L., Subramaniyan B. (2019). PBPK modeling-based optimization of site-specific chemo-photodynamic therapy with far-red light-activatable paclitaxel prodrug. *Journal of Controlled Release*.

[16] Lucky S. S., Soo K. C., Zhang Y. (2015). Nanoparticles in photodynamic therapy. *Chemical Reviews*.

[17] Farrer N. J., Salassa L., Sadler P. J. (2009). Photoactivated chemotherapy (PACT): the potential of excited-state d-block metals in medicine. *Dalton Transactions*.

[18] Zhang Q., Kuang G., He S. (2020). Photoactivatable prodrug-backboned polymeric nanoparticles for efficient light-controlled gene delivery and synergistic treatment of platinum-resistant ovarian cancer. *Nano Letters*.

[19] Dell’Anna M. M., Censi V., Carrozzini B. (2016). Triphenylphosphane Pt(II) complexes containing biologically active natural polyphenols: synthesis, crystal structure, molecular modeling and cytotoxic studies. *Journal of Inorganic Biochemistry*.

[20] Censi V., Caballero A. B., Pérez-Hernández M. (2019). DNA-binding and in vitro cytotoxic activity of platinum(II) complexes of curcumin and caffeine. *Journal of Inorganic Biochemistry*.

[21] Giordano A., Tommonaro G. (2019). Curcumin and cancer. *Nutrients*.

[22] Rosenberg B., Vancamp L., Trosko J. E., Mansour V. H. (1969). Platinum compounds: a new class of potent antitumour agents. *Nature*.

[23] Dilruba S., Kalayda G. V. (2016). Platinum-based drugs: past, present and future. *Cancer Chemotherapy and Pharmacology*.

[24] Johnstone T. C., Suntharalingam K., Lippard S. J. (2016). The next generation of platinum drugs: targeted Pt(II) agents, nanoparticle delivery, and Pt(IV) prodrugs. *Chemical Reviews*.

[25] Amable L. (2016). Cisplatin resistance and opportunities for precision medicine. *Pharmacological Research*.

[26] Chen S.-H., Chang J.-Y. (2019). New insights into mechanisms of cisplatin resistance: from tumor cell to microenvironment. *International Journal of Molecular Sciences*.

[27] Koon H., Leung A. W. N., Yue K. K. M., Mak N. K. (2006). Photodynamic effect of curcumin on NPC/CNE2 cells. *Journal of Environmental Pathology, Toxicology and Oncology*.

[28] Yallapu M. M., Jaggi M., Chauhan S. C. (2012). Curcumin nanoformulations: a future nanomedicine for cancer. *Drug Discovery Today*.

[29] Wanninger S., Lorenz V., Subhan A., Edelmann F. T. (2015). Metal complexes of curcumin – synthetic strategies, structures and medicinal applications. *Chemical Society Reviews*.

[30] Tsai W. H., Yu K. H., Huang Y. C., Lee C. I. (2018). EGFR-targeted photodynamic therapy by curcumin-encapsulated chitosan/TPP nanoparticles. *International Journal of Nanomedicine*.

[31] Oberoi H. S., Nukolova N. V., Kabanov A. V., Bronich T. K. (2013). Nanocarriers for delivery of platinum anticancer drugs. *Advanced Drug Delivery Reviews*.

[32] Xu X., Ho W., Zhang X., Bertrand N., Farokhzad O. (2015). Cancer nanomedicine: from targeted delivery to combination therapy. *Trends in Molecular Medicine*.

[33] Wang X., Wang X., Guo Z. (2015). Functionalization of platinum complexes for biomedical applications. *Accounts of Chemical Research*.

[34] Santos A. C., Pattekari P., Jesus S., Veiga F., Lvov Y., Ribeiro A. (2015). J. Sonication-assisted layer-by-layer assembly for low solubility drug nanoformulation. *ACS Applied Materials & Interfaces*.

[35] Vergaro V., Papadia P., Petrini P. (2017). Nanostructured polysaccharidic microcapsules for intracellular release of cisplatin. *International Journal of Biological Macromolecules*.

[36] Bonnet S. (2018). Why develop photoactivated chemotherapy?. *Dalton Transactions*.

[37] Pampaloni F., Reynaud E. G., Stelzer E. H. K. (2007). The third dimension bridges the gap between cell culture and live tissue. *Nature Reviews Molecular Cell Biology*.

[38] De Castro F., Vergaro V., Benedetti M. (2020). Visible light-activated water-soluble platicur nanocolloids: photocytotoxicity and metabolomics studies in cancer cells. *ACS Applied Bio Materials*.

[39] Parekh G., Pattekari P., Joshi C. (2014). Layer-by-Layer nanoencapsulation of camptothecin with improved activity. *International Journal of Pharmaceutics*.

[40] Elbert D. L., Herbert C. B., Hubbell J. A. (1999). Thin polymer layers formed by polyelectrolyte multilayer techniques on biological surfaces. *Langmuir*.

[41] Picart C., Mutterer J., Richert L. (2002). Molecular basis for the explanation of the exponential growth of polyelectrolyte multilayers. *Proceedings of the National Academy of Sciences*.

[42] Xu L., Pristinski D., Zhuk A., Stoddart C., Ankner J. F., Sukhishvili S. A. (2012). Linear versus exponential growth of weak polyelectrolyte multilayers: correlation with polyelectrolyte complexes. *Macromolecules*.

[43] Mitra K., Gautam S., Kondaiah P., Chakravarty A. R. (2017). Platinum(II) complexes of curcumin showing photocytotoxicity in visible light. *European Journal of Inorganic Chemistry*.

[44] Gibson D. (2009). The mechanism of action of platinum anticancer agents—what do we really know about it?. *Dalton Transactions*.

[45] Mitra K., Patil S., Kondaiah P., Chakravarty A. R. (2015). 2-(Phenylazo) Pyridine platinum(II) catecholates showing photocytotoxicity, nuclear uptake, and glutathione-triggered ligand release. *Inorganic Chemistry*.

[46] Mitra K., Shettar A., Kondaiah P., Chakravarty A. R. (2016). Biotinylated platinum(II) ferrocenylterpyridine complexes for targeted photoinduced cytotoxicity. *Inorganic Chemistry*.

[47] Florea A.-M., Büsselberg D. (2011). Cisplatin as an Anti-tumor Drug: Cellular Mechanisms of Activity, Drug Resistance and Induced Side Effects. *Cancers*.

[48] Popat A., Liu J., Lu G. Q., Qiao S. Z. (2012). A PH-responsive drug delivery system based on chitosan coated mesoporous silica nanoparticles. *Journal of Materials Chemistry*.

[49] Ou H., Cheng T., Zhang Y. (2018). Surface-adaptive zwitterionic nanoparticles for prolonged blood circulation time and enhanced cellular uptake in tumor cells. *Acta Biomaterialia*.

[50] Misra C., Gaur M., Gupta L. N. (2019). Nanotechnology: emerging platform for drug based delivery system in cancer. *Journal of Drug Delivery and Therapeutics*.

[51] Esfahani M. K. M., Alavi S. E., Movahedi F., Alavi F., Akbarzadeh A. (2013). Cytotoxicity of liposomal paclitaxel in breast cancer cell line MCF-7. *Indian Journal of Clinical Biochemistry*.

[52] Alavi S. E., Esfahani M. K. M., Ghassemi S., Akbarzadeh A., Hassanshahi G. (2014). In Vitro evaluation of the efficacy of liposomal and pegylated liposomal hydroxyurea. *Indian Journal of Clinical Biochemistry*.

[53] Mosmann T. (1983). Rapid colorimetric assay for cellular growth and survival: application to proliferation and cytotoxicity assays. *Journal of Immunological Methods*.

[54] Gökçe Kütük S., Gökçe G., Kütük M., Gürses Cila H. E., Nazıroğlu M. (2019). Curcumin enhances cisplatin-induced human laryngeal squamous cancer cell death through activation of TRPM2 channel and mitochondrial oxidative stress. *Scientific Reports*.

[55] Langhans S. A. (2018). Three-dimensional in vitro cell culture models in drug discovery and drug repositioning. *Frontiers in Pharmacology*.

[56] Gong X., Lin C., Cheng J. (2015). Generation of multicellular tumor spheroids with microwell-based agarose scaffolds for drug testing. *PLoS One*.

[57] Xu Y.-Q., Chen W.-R., Tsosie J. K. (2016). Niosome encapsulation of curcumin: characterization and cytotoxic effect on ovarian cancer cells. *Journal of Nanomaterials*.

[58] Zhang J., Li J., Shi Z. (2017). PH-sensitive polymeric nanoparticles for Co-delivery of doxorubicin and curcumin to treat cancer via enhanced pro-apoptotic and anti-angiogenic activities. *Acta Biomaterialia*.

[59] Schneider C., Gordon O. N., Edwards R. L., Luis P. B. (2015). Degradation of curcumin: from mechanism to biological implications. *Journal of Agricultural and Food Chemistry*.

[60] Naksuriya O., van Steenbergen M. J., Torano J. S., Okonogi S., Hennin W. E. (2016). A kinetic degradation study of curcumin in its free form and loaded in polymeric micelles. *The AAPS Journal*.

[61] Minchinton A. I., Tannock I. F. (2006). Drug penetration in solid tumours. *Nature Reviews Cancer*.

[62] Bryce N. S., Zhang J. Z., Whan R. M., Yamamoto N., Hambley T. W. (2009). Accumulation of an anthraquinone and its platinum complexes in cancer cell spheroids: the effect of charge on drug distribution in solid tumour models. *Chemical Communications*.

[63] Goodman T. T., Ng C. P., Pun S. H. (2008). 3-D tissue culture systems for the evaluation and optimization of nanoparticle-based drug carriers. *Bioconjugate Chemistry*.

[64] Hall M. D., Martin C., Ferguson D. J. P., Phillips R. M., Hambley T. W., Callaghan R. (2004). Comparative efficacy of novel platinum(IV) compounds with established chemotherapeutic drugs in solid tumour models. *Biochemical Pharmacology*.

[65] Oh N., Park J.-H. (2014). Endocytosis and exocytosis of nanoparticles in mammalian cells. *International Journal of Nanomedicine*.

[66] Fortuni B., Inose T., Ricci M. (2019). Polymeric engineering of nanoparticles for highly efficient multifunctional drug delivery systems. *Scientific Reports*.

[67] Senapati S., Mahanta A. K., Kumar S., Maiti P. (2018). Controlled drug delivery vehicles for cancer treatment and their performance. *Signal Transduction and Targeted Therapy*.

