# Supplementary material for: Low-Intensity Light-Responsive Anticancer Activity of Platinum(II) Complex Nanocolloids on 2D and 3D In Vitro Cancer Cell Model
Source: Bioinorg Chem Appl. 2022 Apr 23;2022:9571217. doi: 10.1155/2022/9571217 (PMC9056248; doi:10.1155/2022/9571217)
Supplement: Supplementary Materials — The supplementary file contains S1 figure with Nanocolloids size and Zeta potential measurements; S2 figure with Release kinetics; S3 figure with Cytotoxicity test performed on MCF 7 cell lines; S4 figure with Optical representative images of spheroids of MCF 7 cell lines; S5 figure with Confocal Z-stack micrographs of MCF7 treated with Curcumin (50 μM) and Pt-Curc NCs (50 μM) in the dark for 2 hours. [file 9571217.f1.docx]

Low intensity light-responsive anticancer activity of Platinum(II) complex nanocolloids on

2D and 3D *in vitro* cancer cell model

Viviana Vergaro,*^1^ Francesca Baldassarre,^1,2^ Federica De Castro,^2^ Danilo Migoni,^2^ Maria Michela Dell’Anna,^3^ Piero Mastrorilli,^3^ Francesco Paolo Fanizzi^2^ and Giuseppe Ciccarella*^1,2^

^1.^ Institute of Nanotechnology, CNR NANOTEC, Consiglio Nazionale delle Ricerche, Via Monteroni, 73100 Lecce, Italy

^2.^ Biological and Environmental Sciences Department, UdR INSTM of Lecce University of Salento, Via Monteroni, 73100 Lecce, Italy.

^3.^ DICATECh, Politecnico di Bari, via Orabona, 4, 70125 Bari, Italy

* correspondig author: [viviana.vergaro@nanotec.cnr.it](mailto:viviana.vergaro@nanotec.cnr.it) and [giuseppe.ciccarella@unisalento.it](mailto:giuseppe.ciccarella@unisalento.it)

**Figure S1**: Nanocolloids size and Zeta potential measurements

**Figure S2:** Release kinetics

**Figure S3:** Cytotoxicity test performed on MCF 7 cell lines.

**Figure S4:** Optical representative images of spheroids of MCF 7 cell lines

**Figure S5:** Confocal Z-stack micrographs of MCF7 treated with Curcumin (50 μM) and Pt-Curc NCs (50 μM) in the dark for 2 hours.


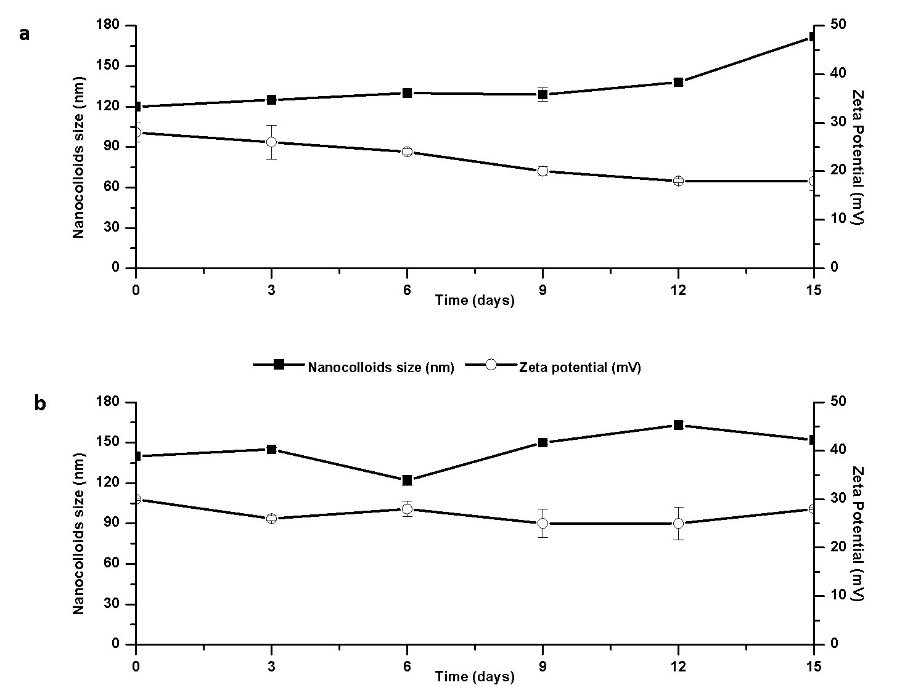


**Figure S1**: Nanocolloids size and Zeta potential measurements of Pt-Curc complex prepared with (a) chitosan only and with (b) (CHI/PEC)_2.5_


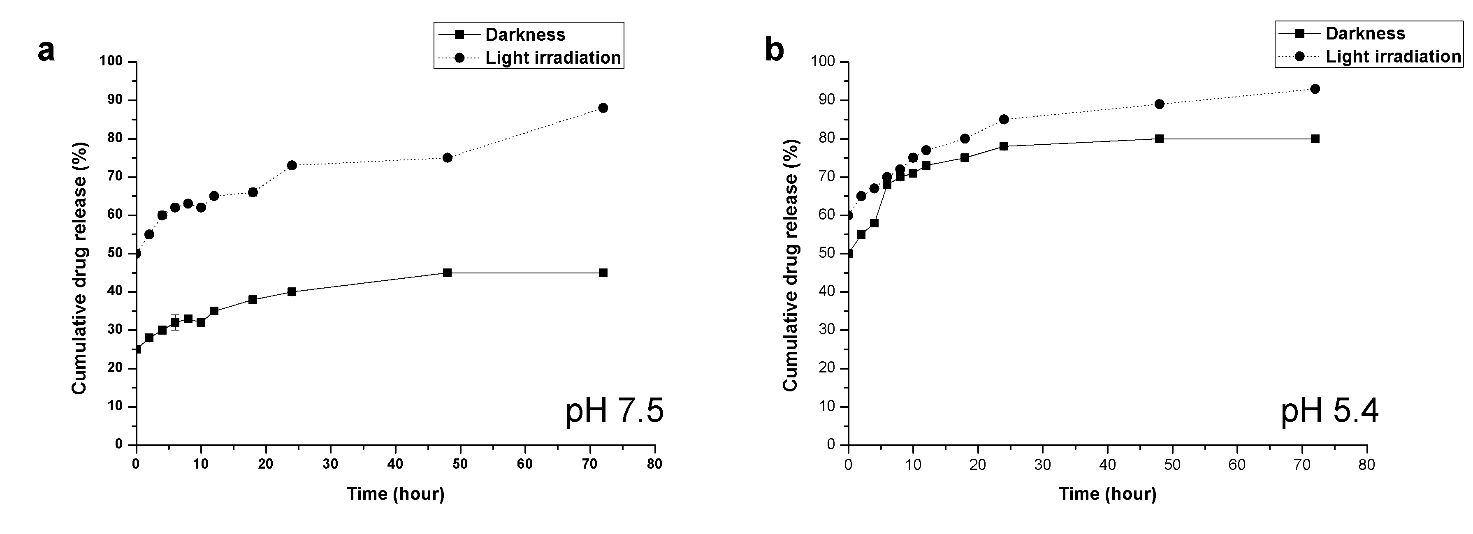


**Figure S2**: Release kinetics of Platinum from Pt-Curc NC (CHI/PEC)_2.5_ under physiological (a) and acidic pH (b).


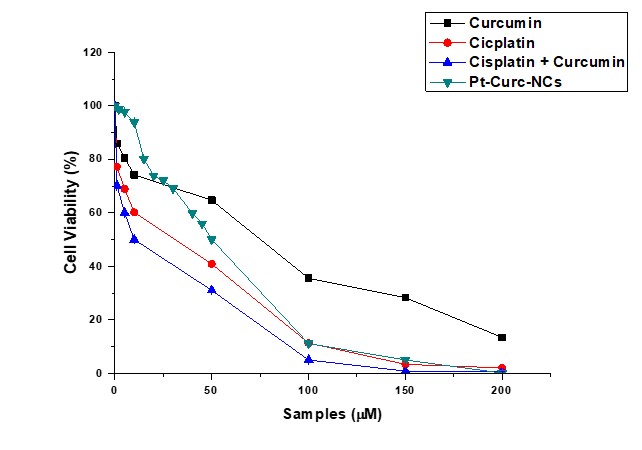


**Figure S3:** Cytotoxicity test performed on MCF 7 cell lines.


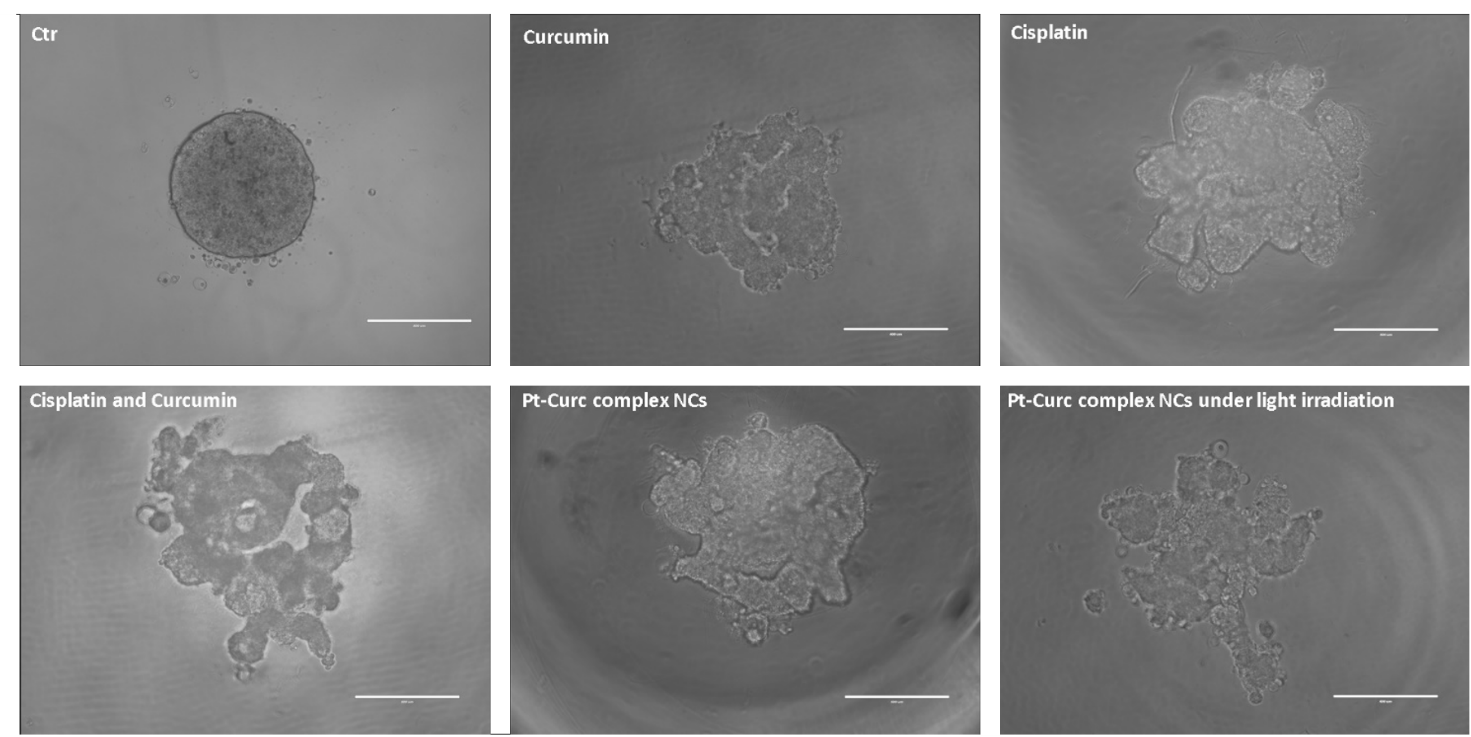


**Figure S4:** Optical representative images of spheroids of MCF7 cell lines forming spontaneously in 50 μL medium containing 4·10^3^ cells after 24 h. Size marker = 400 μm.


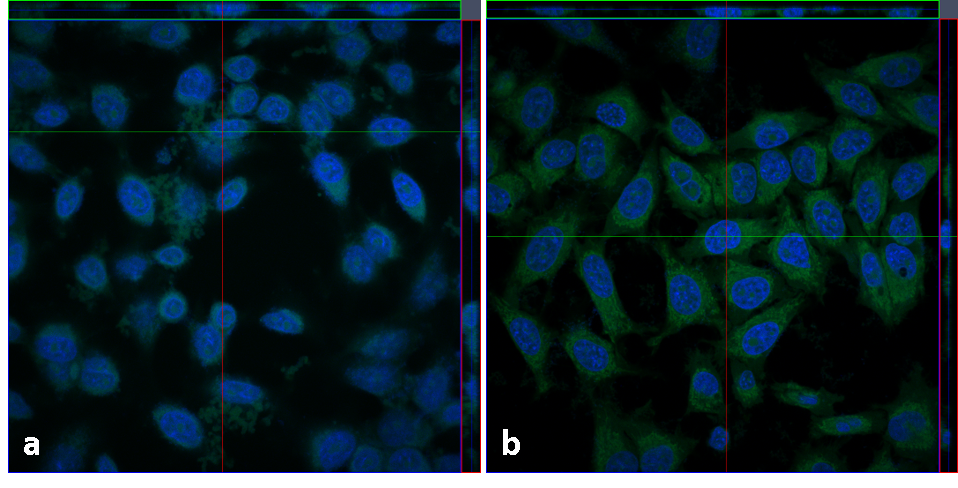


**Figure S5:** Confocal Z-stack micrographs of MCF7 treated with Curcumin (50 μM) and Pt-Curc NCs (50 μM) in the dark for 2 hours.
